# Maternal Circulating Levels of Activin A, Inhibin A, sFlt-1 and Endoglin at Parturition in Normal Pregnancy and Pre-Eclampsia

**DOI:** 10.1371/journal.pone.0004453

**Published:** 2009-02-11

**Authors:** Aparna Reddy, Sangeeta Suri, Ian L. Sargent, Christopher W. G. Redman, Shanthi Muttukrishna

**Affiliations:** 1 Fetal Medicine Unit, Elizabeth Gareth Anderson Hospital, University College Hospital, London, United Kingdom; 2 Elizabeth Gareth Anderson UCL Institute for Women's Health, London, United Kingdom; 3 Nuffield Department of Obstetrics and Gynaecology, University of Oxford, John Radcliffe Hospital, Oxford, United Kingdom; National Institute of Child Health and Human Development/National Institutes of Health, United States of America

## Abstract

**Background:**

Maternal circulating levels of anti-angiogenic factors such as soluble fms-like tyrosine kinase-1 (sFlt-1), endoglin (sEng) and placental proteins like activin A and inhibin A are increased before the onset of pre-eclampsia. There is evidence for oxidative stress in pre eclampsia. Recently it was shown that placental oxygen concentration is related to sFlt-1 and inhibin A. In addition it is reported that oxidative stress markers are increased in placental tissue delivered after labour. Therefore, the objective of this study is to investigate if these proteins are altered in maternal circulation of labouring pre-eclampsia and normal pregnancies.

**Methodology:**

To assess the effects of labour, samples were taken from 10 normal pregnant (NP) and 10 pre-eclamptic (PE) women pre-labour, full dilation, placental delivery and 24 h. To assess the effects of placental delivery, plasma samples were taken from 10NP and 10PE women undergoing elective Caesarean section, pre-delivery, placental delivery and 10 min, 60 min and 24 h post delivery. SFlt-1 and sEng and activin A and inhibin A were measured using commercial and in house ELISA's respectively.

**Results:**

The levels of sFlt-1 and sEng were significantly higher in PE compared to NP women in both groups. In labour, sFlt-1 levels increased significantly at full dilatation in PE women, before declining by 24 hr. However there was no significant rise in sEng levels in labour. Activin A and inhibin A levels declined rapidly with placental delivery in NP and PE pregnancies. There was a significant rise in activin A levels during labour in PE compared to pre labour, but inhibin levels did not increase.

**Conclusion:**

Labour in pre-eclamptic women increases the levels of sFlt-1 and activin A. This pilot data suggests that increase in the maternal levels of these factors in labour could predict and/or contribute to the maternal syndrome postpartum.

## Introduction

Pre-eclampsia is a pregnancy specific complication and a major cause of maternal and fetal morbidity and mortality. Early stages of placental development occur under relatively hypoxic conditions [1] that favour invasive cytotrophoblast proliferation [2]. Invading cytotrophoblasts are pivotal in the transformation of the maternal uterine circulation. In normal pregnancies, the transformation of the spiral arteries is completed around mid gestation in order to optimise the distribution of maternal blood into low resistance uterine vascular network and ultimately inside the placental intervillous chamber [3].

In pre-eclampsia, there is defective early trophoblastic invasion of the decidua due to failure of conversion of the spiral arteries into low-resistance channels. This results in retention of vasoreactivity within the placental vascular bed which causes diminished and variable perfusion to the intervillous space [4]–[6]. The magnitude of defective trophoblastic invasion of the spiral arteries correlates with the severity of the hypertensive disorder [7]. The mechanism that leads to the invasion and transformation of the spiral arteries by trophoblasts in normal pregnancy and the factors that restrict trophoblast invasion in pregnancy complications such as pre-eclampsia are poorly understood.

Within the last decade, several studies have investigated possible biochemical markers altered in pre-eclampsia [8]–[9]. TGF beta proteins produced by the placenta and vascular endothelial cells are involved in cell proliferation and differentiation. High concentrations of inhibin A and activin A are found in the maternal circulation and the placenta is thought to be the predominant source [10]–[12], although activin A is also produced by peripheral mononuclear cells and vascular endothelial cells [13]. In women with established pre eclampsia, circulating levels of inhibin A and activin A are significantly elevated compared to normal pregnancy [12] and studies have shown serum levels can be raised as early as 10–15 weeks of pregnancy in women who subsequently develop pre-eclampsia [14].

More recently, pro and anti angiogenic proteins have been investigated in maternal circulation of pre-eclampsia patients [15]. Vascular endothelial growth factor (VEGF) is an important angiogenic protein in pregnancy. Placental growth factor (PlGF) is a placental protein that potentiates the effect of VEGF [16]. Soluble fms-like tyrosine kinase 1 (sFlt-1), also known as soluble vascular endothelial growth factor (VEGF) receptor1 is an anti-angiogenic protein that is increased prior to the onset of the clinical disease in women with pre-eclampsia. sFlt-1 levels correlate with the severity and the time to the onset of the disease [15]–[18]. Soluble Endoglin (sEng) is a novel anti-angiogenic protein. It is a cell surface coreceptor for transforming growth factor -β1 and -β3 isoforms and is expressed highly in endothelial cells and the trophoblast [19]. Its levels have been shown to rise in the last two months of normal pregnancy, however the levels rise earlier and more steeply in women who go on to develop pre-eclampsia, reaching a peak at the onset of clinical disease[15]–[16], [20]–[21].

In pre-eclampsia, there is increased oxidative stress and maternal systemic inflammation [22]–[23]. The mechanism behind the increases in these hormones is unknown, although placental hypoxia has been shown to alter activin A and sFlt-1 levels in vitro [24]–[25]. Recently it has been shown that oxidative stress stimulates activin A production and secretion from placental explants and endothelial cells and that in women with pre-eclampsia, circulating levels of activin A are significantly associated with markers for excessive oxidative stress [26]. At parturition, activin A has also been shown to be increased in labour compared to an elective Caesarean section at term [27].

An aspect of pre-eclampsia which is intriguing is the development of hypertension postpartum. Hypertension arising de novo in the postpartum period in women who did not have hypertension in the antepartum period could be a non-specific phenomenon in women with no underlying disease. However, it could also be late onset pre-eclampsia or the unmasking of underlying essential hypertension. The British Eclampsia Survey [28] reported an incidence of 18% intrapartum, and 44% postpartum eclampsia. The majority of postpartum eclamptic fits occurred within the first 48 hours after delivery. Only 12% presented after this period and indeed only 2% of cases occurred after seven days postpartum. Another severe form of pre-eclampsia is HELLP syndrome and a third of these cases are diagnosed for the first time postpartum [29]. These cases are also known to worsen postpartum prior to clinical improvement.

The fact that pre-eclampsia and eclampsia can occur in the postnatal period raises the question as to the role of placental factors which could predict and/or contribute to the maternal syndrome postpartum. The objective of this study was to investigate the effect of the labour and the removal of the placenta on the circulating concentrations of inhibin A, activin A, sFLT-1 and sEng. In addition, we studied the relative contributions of these proteins in pre-eclampsia and normal pregnant women as the release of these molecules could be a potential trigger to postpartum worsening of pre-eclampsia.

## Materials and Methods

### Subjects

To investigate the effect of labour, patients undergoing induction of labour were recruited (Table-1). They were diagnosed by new hypertension in pregnancy (blood pressure ≥140/90 mmHg, on at least two occasions at least six hours apart) and new proteinuria (≥2+ on dipstick testing on at least two occasions or ≥500 mg protein in a 24 hour urine collection, in the proven absence of a urinary tract infection). Peripheral venous blood samples were taken [1] before induction of labour, [2] at full dilatation confirmed by a vaginal examination by the midwife or by visible signs of onset of second stage, [3] immediately after placental delivery and [4] 24 hours after delivery. All patients recruited in this group (10 normal pregnant and 10 pre-eclampsia women) delivered vaginally.

**Table 1:** Labour study.

Patient characteristics	Normal pregnant	Pre-eclamptic women	P value
	N = 10	N = 10	Mann Whitney U test
**Maternal age (years)**	30 (4.9)	32 (4.9)	Ns
**Nulliparity (%)**	50	80	-
**Gestation at sampling/delivery (weeks)**	41 (1.6)	37 (1.4)	<0.001
**BMI**	23.9(0.9)	29.04(1.8)	<0.05
**Smoking**	none	none	-
**Diastolic BP at sampling (mmHg)**	73(5.2)	93 (16.0)	<0.01
**Systolic BP at sampling (mmHg)**	120 (8.2)	140 (19.6)	<0.001
**Maximum diastolic BP (mmHg)**	84 (6.2)	111 (9.1)	0.001
**Maximum systolic BP (mmHg)**	133 (7.3)	174 (13.0)	0.0001
**Maximum 24 hr proteinuria (gms)**	Not available	881(510–3027)	-
**Birthweight (g)**	3606 (456)	2626 (525)	0.001
**Placental weight (g)**	682 (115)	612 (144.4)	0.05
**SGA (Birth weight <10^th^ centile for gestation)**	none	40%	-

To investigate the effects of placental separation alone, ten pre-eclamptic women scheduled for Caesarean section were recruited from hospital in-patients (Table-2). Ten healthy pregnant women scheduled for elective Caesarean section for breech presentation or previous Caesarean section in the third trimester of pregnancy were recruited as controls. They had no significant past medical history, current illness or used regular medication. Peripheral venous blood samples were taken [1] prior to Caesarean section, [2] at the time of placental delivery, [3] 10 min post delivery, [4] 60 min post delivery and [5] 24 hour post placental delivery.

**Table 2:** Elective Caesarean Section study.

Patient characteristics	Normal pregnant	Pre-eclamptic women	P value
	N = 10	N = 10	Mann Whitney U test
**Maternal age (years)**	33 (5.7)	34 (5.3)	Ns
**Nulliparity (%)**	10	80	-
Gestation at sampling/delivery (weeks) **	39 (0.5)	35 (4.3)	<0.01
**BMI**	25.2(0.7)	26.6(1.9)	ns
**Smoking**	none	none	-
**Diastolic BP at sampling (mmHg)**	63(9.4)	96 (10.13)	<0.01
**Systolic BP at sampling (mmHg)**	118(13.6)	153(21.3)	<0.001
**Maximum diastolic BP (mmHg)**	80 (7.7)	117 (10.13)	<0.001
**Maximum systolic BP (mmHg)**	133 (7.6)	179(17.9)	<0.001
**Maximum 24 hr proteinuria (gms)**	Not available	3541(739–18293)	-
**Birth weight (g)**	3624 (445.5)	2024 (831)	<0.01
**Placental weight(g)**	714 (164)	468(182.5)	<0.05
**SGA (Birth weight <10^th^ centile for gestation)**	none	60%	-

Written consent was obtained from each woman after receiving full written information about the research project. This study was approved by the OxREC (Oxfordshire Clinical Research Ethics Committee).

### Measurement of Activin A

Total Activin A (follistatin bound and unbound) was measured using a two-site ELISA as previously described [13]. Affinity-purified human activin A was used as the assay standard. The detection limit of the assay for purified human activin A was 50 ng/ml. Intra- and inter assay coefficients of variation were 9% and 10%, respectively.

### Measurement of Inhibin A

Dimeric inhibin A was measured using a two-site ELISA described previously [13]. The sensitivity of the assay was 2 pg/ml and the intra- and inter assay variations were 5.2% and 6.5%, respectively.

### sEndoglin and sFlt-1 ELISA

Commercial assays from R&D systems (Abingdon, Oxford, UK) were used to measure sFlt-1 (kits) and soluble Endoglin (Duo sets). All samples were assayed in duplicate. The minimum detection limit of the sFlt-1 ELISA was 31.3 ng/ml and sEng was 62.5 ng/ml. Inter and intra assay variations for both assays were <12%.

### Statistical analysis

Non parametric tests were carried out as data were not normally distributed. To analyse the effect of labour and placental separation at Caesarean section on the release of activin A, Inhibin A, sFlt-1-1 and sEng, the Mann-Whitney U test was used for analysis to compare the unpaired data and Wilcoxon test for the paired data. Data expressed as median (ranges). Significance set at P<0.05. Statistical analyses were carried out using GraphPad Prism version 4.0 (GraphPad Inc., San Diego, CA).

## Results

The clinical characteristics of each group are presented in Table 1. There were no significant differences between the two groups in terms of age or parity, but there was a significant difference in the gestation at delivery as patients with pre-eclampsia delivered early compared to normal pregnant women. All maternal notes were reviewed to check for smoking status and none of the women were known smokers. Maximum blood pressure and maximum proteinuria were significantly increased in the pre-eclampsia group compared to the normal pregnant group. The median duration of the first stage of labours (ie onset of labour until full dilatation) varied between 340 min (130–900 range) for the normal pregnant group and 188 min (90–645) for the pre-eclampsia group. However this was not statistically significant.

### Maternal serum activin A

**Figure 1:**
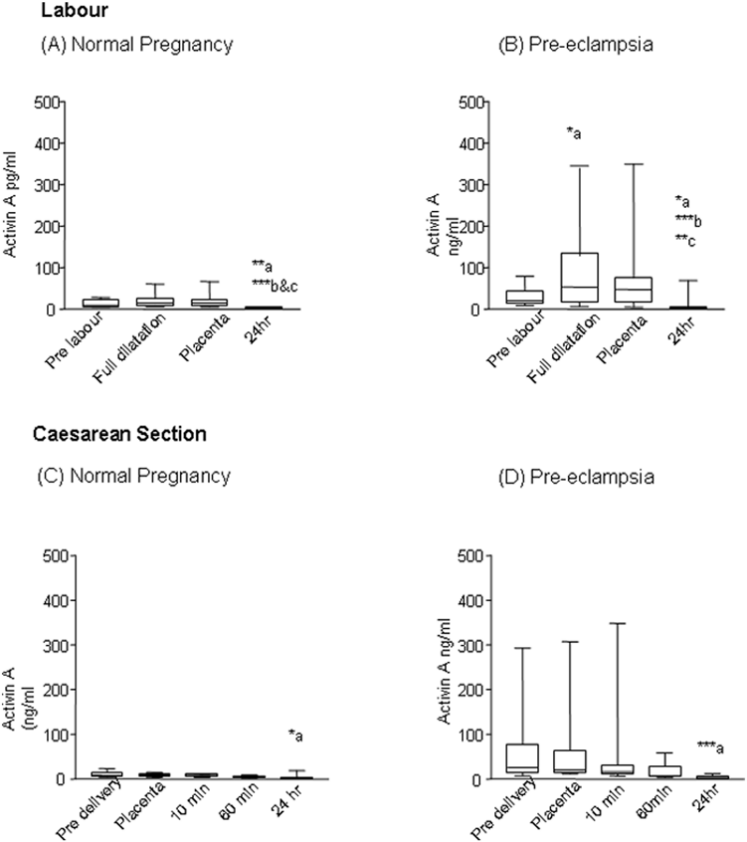
Activin A levels (median (ranges) ng/ml) in maternal plasma during labour (A,B) and Caesarean section (C,D) in normal pregnancy (n = 10) and women with pre-eclampsia (n = 10). In normal pregnancy labour (A) there was a significant decline in activin A levels by 24 hr (a**p<0.01, pre-labour vs 24 hr, b ***p<0.001-full dilatation vs 24 hr, and c***p<0.001-placental delivery vs 24 hr. In pre-eclampsia labour (B) there was a significant increase in levels of activin A, (a*p<0.05 pre-labour vs full dilatation) and a significant decline postpartum (a*p<0.05 pre-labour vs 24 hr, b***p<0.001-full dilatation vs 24 hr, and c**p<0.01-placental delivery vs 24 hr). At Caesarean section a significant decline in levels of activin A by 24 hr was noted with placental delivery in normal pregnancy and pre-eclampsia (C,D). (NP a*p<0.05 and PE, a ***p<0.001 placental delivery vs 24 hr).

In the labouring group, the pre-labour values were significantly higher in pre-eclampsia (19.8(8.3–79)) women compared to the controls (8.8(4.6–28)), (Fig 1A & 1B). In normal pregnancy, during labour there was no significant increase compared to pre-labour levels of activin A at full dilatation; however the levels declined rapidly within 24 hours of delivery (2.5(1.06–4.8)). In pre-eclampsia, there was a significant increase with full dilatation compared to pre-labour (265%, P<0.05) and with placental delivery the levels rapidly declined to 3.2(0.9–68.5)after 24 hr of delivery (full dilatation vs 24 hr p<0.001).

The plasma levels of activin A in the Caesarean section group are shown in Fig 1C and D. The levels of activin A in pre-eclamptic women were significantly higher in pre-delivery samples compared to normal pregnant (25(7.3–29.2) ng/ml vs 8.3 (4.2–23.4); p<0.01 respectively). With placental separation, the levels of circulating activin A declined rapidly and dropped significantly after 24 hours in normal pregnancy to 1.8(0.9–19) ng/ml (∼80%, p<0.01) and in pre-eclampsia declining to 2.4(1.2–11.9)ng/ml(88%, p<0.01), suggesting the placenta is the major source of increased levels of activin A in pre eclamptic women.

### Maternal serum inhibin A

**Figure 2:**
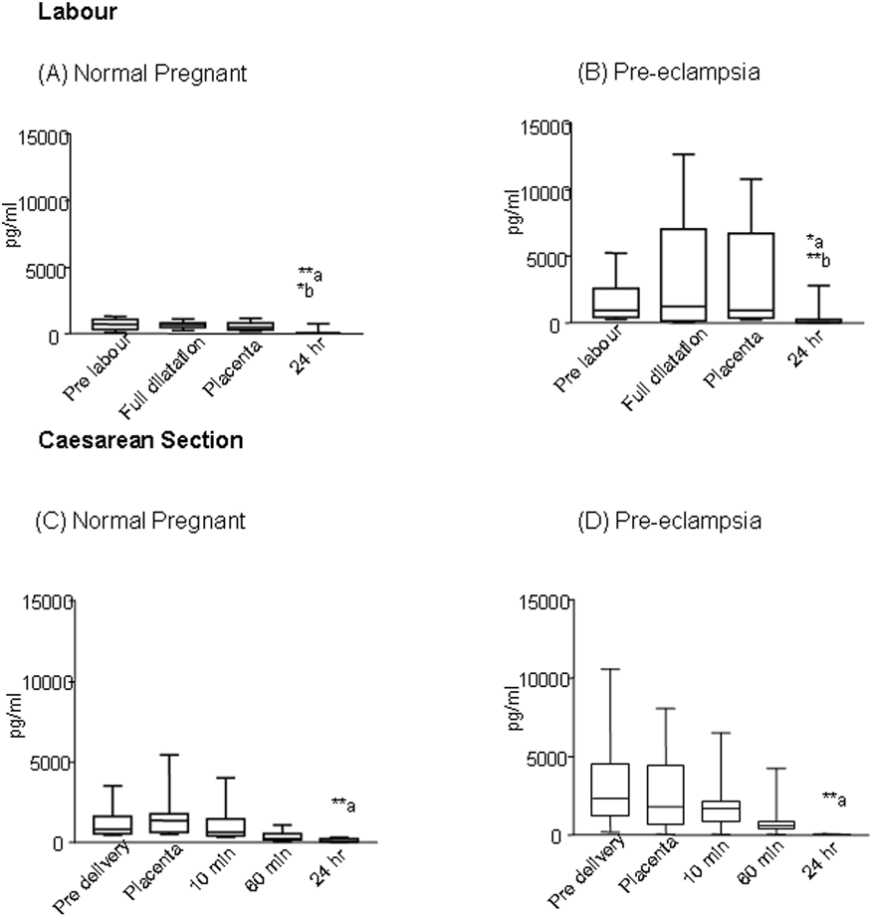
Inhibin A levels (n = 10) in maternal plasma during labour and Caesarean section. Median and ranges for inhibin A levels pg/ml in labour (A, B). In normal pregnancy labour (A) there was a significant decline in inhibin A levels by 24 h (a**p<0.01-prelabour vs 24 hr, full dilatation vs 24 hr, and b* p<0.05-placental delivery vs 24 hr. A similar decline was present in PE labour (B) (a*p<0.05, a-pre-labour vs 24 hr, b **p<0.01-placental delivery vs 24 hr), but the increase noted between pre-labour and full dilatation in pre-eclamptic women was not significant. At Caesarean section (C,D) a significant decline in levels of inhibin A by 24 hr was noted with placental delivery. (NP and PE a**p<0.01 placental delivery vs 24 hr).

In the labouring group, the pre-labour values of inhibin A in pre-eclampsia were (944.7 (272–5274) pg/ml) women compared to normal pregnancy (697.5 (78–1305) pg/ml) (figure 2A & B). During labour, there was no increase in plasma levels in normal pregnant women but in pre-eclampsia women, inhibin A levels at full dilatation (1240 (39–12669) pg/ml) were higher compared to pre-delivery values (944.7 (272–5274) pg/ml), although this increase did not reach statistical significance. Following placental delivery, the levels rapidly declined after 24 hr in normal pregnancy (p<0.01) and pre-eclamptic women (p<0.01). Inhibin A measured pre-delivery in the Caesarean section sub group of study was twofold higher (p<0.05) in pre-eclamptic women (2372 (244–10574) pg/ml) compared to normal pregnant (821(452–3523) pg/ml). The levels declined with placental delivery and after 24 hours declined further to 62.5(39–3191) (∼95%, p<0.01) in normal pregnancy and) in pre-eclamptic women to 39(39–63) pg/ml (98%, p<0.01 (Fig 2C & D).

### Maternal serum soluble Flt-1

**Figure 3:**
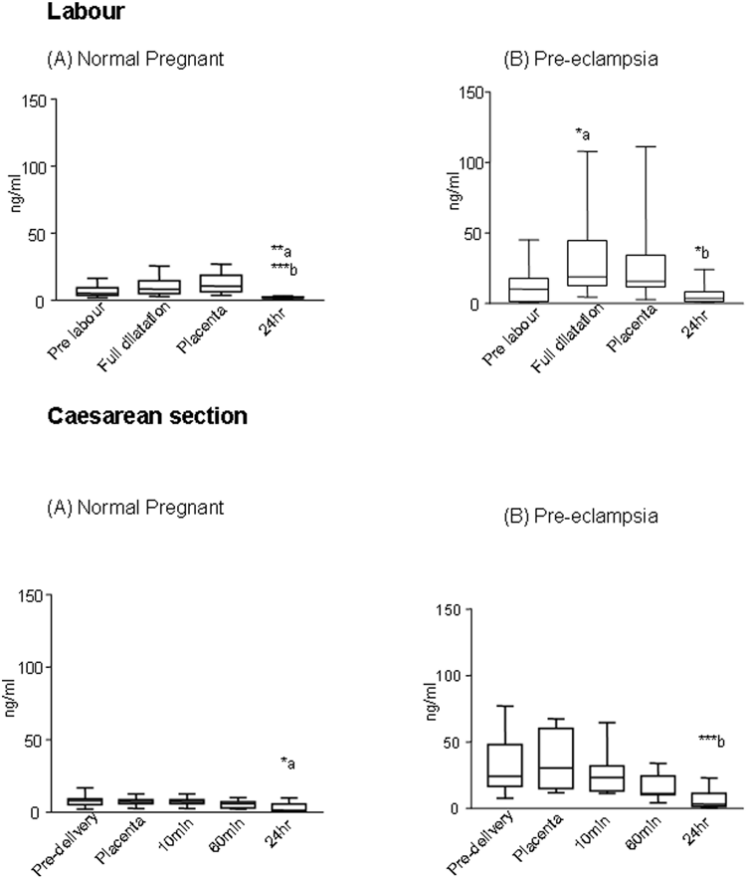
Median and ranges (n = 10) for sFlt-1 levels (ng/ml) in maternal plasma during labour (A,B) and Caesarean section (C,D) in normal pregnancy and women with pre-eclampsia. In normal pregnancy labour (A) the levels showed a significant decline by 24 hr (a**p<0.01-full dilatation vs 24 hr, and b***p<0.001-placental delivery vs 24 hr). In pre-eclampsia labour (B) a significant increase in sFlt-1 levels was noted (a*p<0.05, -pre-labour vs full dilatation) and the levels declined post-delivery (b*p<0.05-full dilatation vs 24 hr). At Caesarean section (C,D) a significant decline in levels of sFlt-1 by 24 hr was noted compared to those at placental delivery (NP a*p<0.05 and PE b***p<0.001, placental delivery vs 24 hr).

The level of sFlt-1in the pre-labour samples of pre-eclamptic women (10.1 (0.6–45) ng/ml) in the labour group was nearly two-fold higher (p<0.05) than in normal pregnancy (4.9 (1.6–16.2) ng/ml). In normal pregnancy, during labour, (Fig 3A) there was no significant change in sFlt-1 levels at full dilatation, although an increasing trend was noted it probably did not achieve statistical significance due to small sample size SFlt-1 levels significantly decreased after 24 hours (82% p<0.007) of delivery. However, in pre-eclamptic women, the levels of plasma sFlt-1 increased during labour (∼180%, p<0.05) and significantly (80%, p<0.05) declined after 24 hr (Fig 3B).

The levels of plasma sFlt-1 were 3 times higher in the pre-delivery samples of pre-eclamptic women(24.3 (7.6–77) ng/ml) in the Caesarean section group compared to normal pregnancy pre-delivery values of 7.9 (2.1–16.8) ng/ml (p<0.001). Furthermore, the pre-delivery levels in women with pre-eclampsia in the Caesarean section group at 34 weeks were 24.3 (7.6–77) ng/ml compared to the levels of 10.1 (0.6–45) ng/ml in the pre-eclamptic women of the labour group who were 37 weeks gestation (p = 0.004). In this cohort of the study in both normal and pre-eclamptic women the levels declined rapidly (NP 80%, P<0.05, PE ∼90%,p<0.001) following delivery and a further decline in levels was observed 24 hrs post delivery (Fig 3C & D).

### Maternal serum sEndoglin

**Figure 4:**
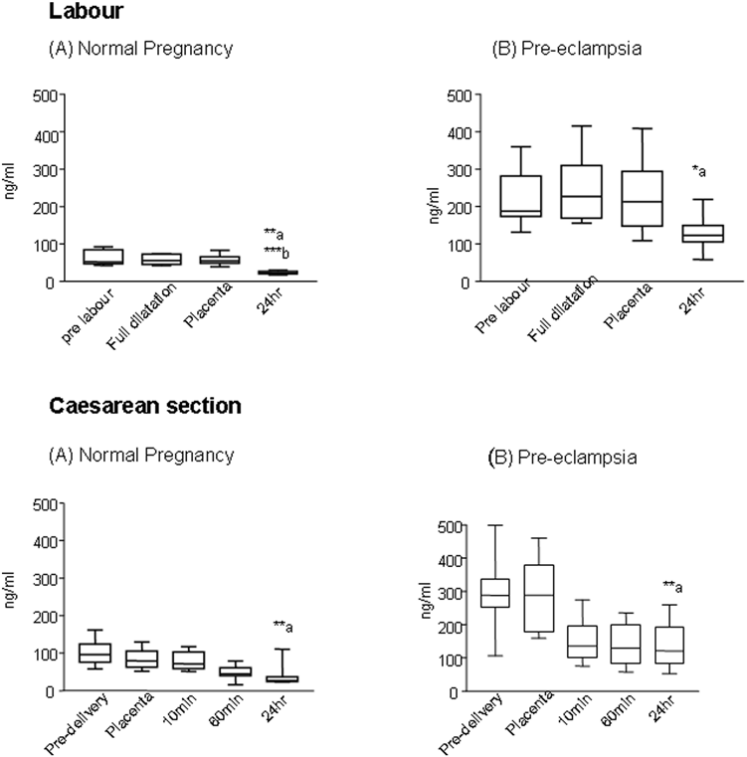
sEndoglin levels in maternal plasma during labour (A, B) and Caesarean section (C,D). Median and ranges for sEndoglin ng/ml. In normal pregnancy labour (A) the levels declined by 24 hr (a***p<0.001, pre-labour vs 24 hr, b**p<0.01-full dilatation vs 24 hr, and placental delivery vs 24 hr). In pre-eclampsia labour (B) a similar decline by 24 h was noted (a*p<0.05, pre-labour vs 24 hr, full dilatation vs 24 hr, and placental delivery vs 24 hr). At Caesarean section (C,D) a significant decline in levels of sEndoglin by 24 hr was noted with placental delivery in both normal pregnancy and pre-eclampsia (a**p<0.01).

The pre-labour values of sEndoglin levels in the labour group were three fold higher in pre-eclamptic women (188 (131–359)ng/ml) compared to normal pregnancy (52.2 (43–92) ng/ml) prior to start of labour (P<0.0001, figure 4A & B). During labour, there was no increase in plasma levels in normal pregnant women or in the pre-eclamptic women. Following placental delivery, the levels rapidly declined after 24 hrs to 22.2 (17.5–30) ng/ml normal pregnancy (p<0.01) and to 123 (57–219) ng/ml in pre-eclamptic women (p<0.01).

sEndoglin measured pre-delivery in the Caesarean section sub group of study was three fold higher in pre-eclamptic (288(106–499)) compared to 96.4 (58–161) ng/ml in normal pregnant women (p<0.001). The levels declined with placental delivery and by 24 hours declined by ∼65% to 27 (22–110)ng/ml, (p<0.01) in normal pregnancy, and by ∼55% to 120(52–259)ng/ml, (p<0.01) in pre-eclamptic women suggesting extra placental source(s) for the increased levels of sEng in pre-eclampsia (Fig 4C and D).

## Discussion

This study confirms that maternal circulating levels of activin A, inhibin A, sFlt-1 and sEng are significantly higher in pre-eclampsia patients compared to normal control pregnant women, consistent with other reports [12], [14], [28]–[29], [16], [19]. This is the first study to report the changes in these TGF beta proteins at parturition in pre-eclamptic patients to study if the process of labour affects these proteins in maternal circulation. Interestingly, in labour at full dilatation pre-eclamptic patients had significantly higher levels of activin A and sFlt-1 compared to pre-labour levels, suggesting the process of labour and/or induction of labour may increase maternal circulating levels of these proteins in pre-eclampsia. Activin A levels were higher in labour compared to Caesarean section in normal pregnancy consistent with a previous study [27]. However, sFlt-1 levels were not altered in the normal pregnant women in labour indicating that these changes are specific to patients with pre-eclampsia. Inhibin A and sEng levels were not significantly altered in both groups in labour.

The levels of activin A and inhibin A were higher in the plasma of pre-eclamptic women who had a Caesarean section around 34 weeks, compared to the pre-delivery levels in the pre-elamptic women in whom labour was induced. This finding is consistent with our previous observation that patients with early onset pre-eclampsia have higher levels of inhibin A and activin A [14]. In the Caesarean section subgroup of the study, levels of activin A and inhibin A declined rapidly post delivery in normal pregnancy (∼80%) and preeclampsia (88%). Previous studies have shown that the major source of maternal circulating levels of activin A and inhibin A is the placenta [12]. The rapid decline of the plasma levels of these proteins seen here following placental delivery in both groups confirms the placenta as the main source of these proteins in maternal circulation. Schneider-Kolsky *et al* [32] performed a longitudinal study of nine normal pregnant women undergoing induction of labour and noticed no difference in Activin A levels in labour in a normal pregnancy compared to pre-delivery values. Although the findings were similar in normal labour, in women with pre-eclampsia there was a significant increase in the levels of activin A during labour (pre labour vs full dilatation) and a rapid decline after placental delivery (declined by 90% in 24 hr).

Several studies have reported high levels of circulating sFlt-1 in maternal serum in pre-eclampsia [15], [33]–[35], and it has been speculated to be causal to the maternal endothelial cell damage [17]. In this study, we report for the first time that levels of sFlt-1 increased at full dilatation compared to pre-labour in pre eclampsia. However, in our study, the rate of decline after placental delivery by 24 hr was not different between normal pregnancy and pre eclampsia in Caesarean section (NP 80%, PE 90%) and labour groups (NP 82%, PE 80%) as reported previously – [36]. In the previous study, plasma sFlt-1 levels were measured at some time in the 48 hours post delivery as the percent change of the serum sFlt1 concentration between delivery and postpartum. The values were then adjusted for the hours between the sample collections. The inherent weakness in this study was the assumption that the actual decline post delivery is linear. In our study we show a trend in post delivery decline as samples from all patients were taken at specified time points and more frequently.

Consistent with previous reports, in this study we report elevated levels of sEndoglin in pre-eclampsia [37]. In addition, for the first time we report a rapid decline of this factor after placental delivery. The percentage of decline in sEng in normal pregnancy was ∼65% after 24 hr delivery compared to >55% in pre-eclamptic patients. However, the process of labour does not significantly affect the levels of sEng in pre-eclampsia or normal pregnancy.

We acknowledge that one of the limitations of this study is the inability to match the cases and controls for gestation. This is an intrinsic problem faced by researchers in this field as pre eclamptic women, by the nature of the pathology, are usually delivered before term. However, as the primary aim of this study was to observe the trend of levels of these factors within a cohort, it is less likely that this could bias the results.

We hypothesize that labour as a possible model for a hypoxia reperfusion insult and this is suggested by several indirect evidences. Doppler studies have shown a correlation between uterine artery resistance and the strength of the contractions during labour [39]–[40]. The contractions of labour are known to influence fetal oxygen saturation [40] and in some instances are severe enough to influence the feto-placental circulation [41]. The intermittent periods of hypoxia due to contraction are followed by reperfusion of the tissues, which leads to increased oxygen tension and the generation of reactive oxygen species in the placenta [42]. In addition, evidence of increased oxidative stress markers has been found in laboured placentas, the expression of these markers being related to the duration of labour [43]–[44].

From our study it can be postulated that the levels of hypoxia ischaemia induced by labour are not severe enough to stimulate materno-placental release of activin A and inhibin A release in normal pregnancy. In contrast, in pre-eclamptic women, a condition characterised by chronic placental hypoxia, the contractions of labour could exacerbate the effect of hypoxia leading to an increased release of activin A. However there could be additional pathways which contribute to the plasma levels. Exposure to pro-inflammatory cytokines could be a possible mechanism for the rise in serum activin A in pre-eclampsia [45]. In contrast, inhibin A was stimulated modestly by IL-1b and not by other cytokines, indicating that the mechanisms involved in the rise of activin A and inhibin A in pre-eclampsia are controlled differentially. The contractions of labour in a compromised placenta due to pre-eclampsia could add to the burden of pro-inflammatory cytokines, this could explain the significant rise of activin A compared to inhibin A in pre-eclamptic labour. In addition the increased circulating activin A levels observed in PE may result from both placental and systemic sources, including inflammatory leukocytes and activated endothelium [13]. In vitro studies have shown that oxidative stress, induced by xanthine/xanthine oxidase, stimulates activin A production and secretion from placental explants and endothelial cells [26], and Many *et al* [44], have shown that Xanthine oxidase is elevated in labour. It is possible that the increased oxidative stress in labour could be one of the mechanisms which contribute to the increased levels in pre-eclampsia.

The mechanism of increased sFlt-1 in pre eclampsia is still not known. Although the placenta appears to be the major source of circulating sFlt-1 [46] other sources, such as activated mononuclear cells, may also contribute to the rise in circulating levels [47]. We have some understanding as to how these anti angiogenic proteins (sFlt-1 and sEndoglin) influence the maternal syndrome, but very little information on its effects in the placenta. *In vitro*, cytotrophoblast is known to produce sFlt-1 under reduced oxygen tension [48] and sFlt-1 inhibits trophoblast migration and differentiation [49]. Makris *et al* [25] show that decrease in blood flow to the utero placental compartment in primates leads to development of hypertension and proteinuria and a renal lesion resembling the endotheliosis of pre-eclampsia in the human. These changes were associated with a tenfold increased circulating concentrations of sFlt-1. In our study, the contractions of labour seem to contribute to a three fold increase in sFlt-1 at full dilation compared to pre-labour. The difference in levels noted compared to primates could be due to a possible difference in the mechanisms involved in production and clearance of these proteins.

Pre-eclampsia is also characterized by high circulating concentrations of soluble endoglin, a novel anti angiogenic substance that has been recently shown to synergize with sFlt-1 in the pathogenesis of pre-eclampsia. Excess levels of the two endogenous circulating anti-angiogenic proteins have been postulated to inhibit VEGF and TGF-β1 signalling respectively, contributing to endothelial dysfunction in the vasculature. Although our study did show that the levels of soluble endoglin increased in pre-eclampsia but labour did not lead to enhanced levels. Serum levels of these proteins are a reflection of the production and clearance from different sources. Very little information is available on how sFlt-1 and sEndoglin are cleared from the circulation, although there is some evidence of increased urinary levels possibly due to glomerular leakage which has been noted to be increased in severe compared to mild pre-eclampsia [51].

Cindrova-davies *et al* [52] found increased evidence of oxidative stress, inflammatory cytokines, angiogenic regulators and apoptosis in laboured placentas. They found that this effect was more pronounced in long labours. The increased levels of Activin A and sFlt-1 in labouring women with preeclampsia could contribute to the inflammatory response and endothelial dysfunction. We can speculate that the increased plasma levels of the TGF proteins which are a result of production and clearance, compounded by the oxidative stress noted in labour could sustain the inflammatory response postpartum [53]–[54]. We have previously shown that the contractions in labour leads to increased shedding of placental debris (syncytiotrophoblast microparticles-STBM) [55], possibly due to the hypoxia reperfusion insult of the placenta. The increase of sFlt-1 noted in labour in women with pre-eclampsia was found to positively correlate with the levels of STBM (unpublished observations), suggesting that the shedding mechanisms are related or sFlt-1 is expressed on the STBM. Indeed Sela et al [56] have recently described increased expression of placental sFlt1-14 (variant of sFlt-1), in the syncytial knots in women with pre-eclampsia. Further research to explore these possibilities is currently under investigation in our laboratory.
